# Colorectal cancer and inulin supplementation: the good, the bad, and the unhelpful

**DOI:** 10.1093/gastro/goae058

**Published:** 2024-07-09

**Authors:** Manon Oliero, Ahmed Amine Alaoui, Claire McCartney, Manuela M Santos

**Affiliations:** Nutrition and Microbiome Laboratory, Institut du cancer de Montréal, Centre de Recherche du Centre Hospitalier de l’Université de Montréal (CRCHUM), Montréal, QC, Canada; Nutrition and Microbiome Laboratory, Institut du cancer de Montréal, Centre de Recherche du Centre Hospitalier de l’Université de Montréal (CRCHUM), Montréal, QC, Canada; Department of Medicine, Faculty of Medicine, Université de Montréal, Montréal, QC, Canada; Nutrition and Microbiome Laboratory, Institut du cancer de Montréal, Centre de Recherche du Centre Hospitalier de l’Université de Montréal (CRCHUM), Montréal, QC, Canada; Nutrition and Microbiome Laboratory, Institut du cancer de Montréal, Centre de Recherche du Centre Hospitalier de l’Université de Montréal (CRCHUM), Montréal, QC, Canada; Department of Medicine, Faculty of Medicine, Université de Montréal, Montréal, QC, Canada

**Keywords:** colorectal cancer, inulin, prebiotic, gut microbiota, short-chain fatty acids

## Abstract

The prebiotic inulin has been vaunted for its potential to reduce the risk of colorectal cancer. Inulin fermentation resulting in the production of short-chain fatty acids, primarily butyrate, has been reported to be associated with properties that are beneficial for gut health and has led to an increased consumption of inulin in the Western population through processed food and over-the-counter dietary supplements. However, in clinical trials, there is limited evidence of the efficacy of inulin in preventing colorectal cancer. Moreover, recent data suggest that improper inulin consumption may even be harmful for gastro-intestinal health under certain circumstances. The main objective of this review is to provide insight into the beneficial and potentially detrimental effects of inulin supplementation in the context of colorectal cancer prevention and enhancement of treatment efficacy.

## Introduction

Colorectal cancer (CRC) is the third-most diagnosed and third-most common cause of cancer-related deaths worldwide [1]. CRC develops over a period of 10–20 years [2], with the earliest alteration in the colon being the formation of preneoplastic lesions or aberrant crypt foci (ACF), which may develop into small adenomas, followed by large adenomas with high-grade dysplasia, and eventual progression to invasive carcinomas and metastasis [3]. Only ∼10% of CRC cases are linked to genetic predisposition, of which familial adenomatosis polyposis (FAP) [4] and Lynch Syndrome [5] are the most prevalent hereditary diseases. FAP results from germline mutations in the adenomatous polyposis coli (APC) tumor suppressor gene and FAP patients develop hundreds of polyps in the intestine that, if left untreated, lead to CRC development [6, 7]. Additionally, inflammatory bowel disease (IBD)—a gastro-intestinal disorder including ulcerative colitis and Crohn's disease—is another risk factor associated with CRC (colitis-associated CRC) [8]. Individuals with colitis have a 1.7 times greater risk of developing CRC compared with the general population [9] and the main factors that increase this risk are the severity and extent of the inflammation, as well as the disease duration [8].

The majority of CRC cases are sporadic and due to the accumulation of somatic mutations over a long period of time. Several factors have been reported to be associated with the initiation and progression of sporadic CRC, notably the consumption of a Western-type diet, lack of physical activity, age, heavy alcohol consumption, smoking [10], and, more recently, the presence of an unstable gut microbial community (dysbiosis) [11].

The gut microbiota is the community of microorganisms inhabiting the gastro-intestinal tract, comprising mostly bacteria, in addition to fungi, protozoa, and archaea [12]. The gut microbiota in CRC has a different composition when compared with that of a healthy gut microbiome [13]. Indeed, certain bacterial strains have been reported to be consistently associated with CRC initiation and progression, such as enteropathogenic *Bacteroides fragilis* [14], *Streptococcus gallolyticus* [15], *Enterococcus faecalis* [16], *Fusobacterium nucleatum* [17, 18], and *Escherichia coli* [19]. These bacteria represent potential pathobionts, defined as resident microbes with pathogenic potential [20]. While pathobionts may be harmless to the host under normal conditions, many of them exhibit higher abundance in CRC [21, 22], and some are associated with chronic inflammatory conditions [23]. Overall, the composition of the gut microbiota influences colon carcinogenesis through various mechanisms. These include the regulation of immune responses and inflammation, as well as the production of genotoxins and metabolites such as short-chain fatty acids (SCFAs) and secondary bile acids [24]. Studies even suggest that increased bile acid levels following the consumption of foods that are high in fat is one of the mechanisms linking the Western diet to CRC [25].

The Western diet, characterized by high fat content, refined sugar, and a large proportion of processed food, is most consistently associated with CRC development [26], whereas fiber-rich diets are linked to CRC prevention [27, 28]. The recommended daily fiber intake is 20–35 g per day [27, 29] and both global fiber consumption and CRC rates vary considerably. In North America, daily fiber intake averages 16 g while CRC cases exceed 40 in 100,000 [27, 29]. In contrast, daily fiber intake in many semi-industrialized rural regions of Africa averages 50 g and CRC cases drop to <5 in 100,000 [27, 29].

In light of these differences, an increasing number of studies have highlighted the importance of high dietary fiber intake in lowering the risk of developing CRC [30]. Dietary fiber acts by increasing viscosity and stool bulk, thereby diluting potentially carcinogenic substances, decreasing transit time through the bowel, and promoting fermentation and SCFAs production in the gut [31]. However, questions remain as to whether the consumption of individual fiber supplements, particularly over a prolonged period (>1 month), may help to prevent or stop the progression of colon carcinogenesis. Particular attention has been paid to the potential benefits of prebiotics, which are defined as “selectively fermented ingredients that result in specific changes in the composition and/or activity of the gastro-intestinal microbiota, thus conferring benefit(s) upon host health” [32].

In the realm of CRC prevention, inulin is a prebiotic that has gathered great attention in the last few years. Inulin is a water-soluble, non-digestible, and fermentable polymer of 2–60 fructose units linked via β-(2, 1) glycosidic bonds with a terminal glucose [33]. It is naturally found in high quantities in chicory roots, Jerusalem artichokes, dandelion greens, garlic, leeks, onions, asparagus, and bananas, with chicory roots representing the primary source of purified inulin used in supplements [34]. Given its gel-like consistency and sweet flavor, inulin is widely used as a functional ingredient in industrialized food [35].

While responses to dietary interventions vary considerably between individuals, inulin has consistently invoked broad and predictable changes in the gut microbiome composition and function [36]. A systemic meta-analysis of clinical trials examining the effects of different prebiotics revealed that inulin-type fructans had the greatest positive impact on gut microbiome composition [37]. Due to its wide-ranging effects on the host microbiome composition, function, and production of metabolites, notably SCFAs and bile acids, that have profound impacts on colonic health and the intestinal immune response, inulin may play various roles in the prevention, attenuation, and development of several gastro-intestinal diseases, including IBD and CRC.

The principal aim of this review is to provide an overview of the current knowledge of the beneficial and harmful effects of inulin supplementation derived from animal experiments (Table 1) and clinical trials (Table 2) in the context of CRC.

**Table 1.** Overview of studies on inulin supplementation in animal models

Animal model	Species	Amount and duration	Control group	Outcomes	Effects	Reference
DMH (CRC)	Rat	2% for 10 weeks	HF diet	SCFAs Bile acids Bacterial enzymes	↑ ↓ ↓	Hijova *et al.* 2009 [71]
		5 mg/0.1 mL (oral gavage) for 18 weeks	Control diet	Tumor incidence	No effect	Verma *et al.* 2014 [76]
				Tumor multiplicity	↓	
		5% and 15% for 5 and 10 weeks (short-/long-chained inulin-type fructan)	Control diet	ACF SCFAs	Variable ↑	Poulsen *et al.* 2002 [61]
		80 g/kg for 28 weeks	Control diet	Inflammation *Lactobacilli*	↓ ↑	Hijova *et al.* 2013 [72]
		5% for 3 weeks	Control diet	Apoptotic cells	↑	Hughes *et al.* 2001 [80]
	Mouse	50 mg/kg (in drinking water) for 120 days	LP or HP diet	ACF	↓	Cantero *et al.* 2015 [62]
		50 mg/kg (in drinking water) for 12 weeks	Control diet	ACF	↓	Mauro *et al.* 2013 [63]
		10% for 15 weeks	Control diet	ACF	↓	Gomides *et al.* 2014 [64]
AOM (CRC)	Rat	10% for 17 weeks	Control diet	ACF *Lactobacillus Bifidobacterium* *E. coli*, *S. enterica*	↓ ↑ ↑ ↓	Pattananandecha *et al.* 2016 [65]
		15% for 35 weeks	HF diet cornstarch	Large ACF	↑	Jacobsen *et al.* 2006 [77]
				Tumor number	↓	
		100 g/kg for 32 weeks and 3 days	HF diet	Adenomas SCFAs	↓ ↑	Femia *et al.* 2002 [79]
		5% for 4 weeks	Control diet	ACF	↓	Bolognani *et al.* 2001 [66]
		10% for 11 weeks	Control diet	ACF	↓	Reddy *et al.* 1997 [67]
		5% for 12 weeks	HF diet corn oil	ACF, colonic pH	↓	Rowland *et al.* 1998 [68]
		2.5%, 5%, and 10% for 11 weeks	Control diet	ACF, colonic pH	↓	Verghese *et al.* 2002 [69]
		100 g/kg for 33 weeks	HF diet	IL-10	↑	Roller *et al.* 2004 [73]
	Mouse	5% for 3 weeks	HF diet	DNA damage SCFAs	↓ ↑	Wu *et al.* 2014 [70]
Orthotopic transplantation (CRC)	Mouse	10% for 5 weeks	Control diet	Tumor size Tumor metastasis SCFAs	↓ ↓ ↑	Wang *et al.* 2023 [82]
AOM/DSS (CAC)	Rat	385 mg/day for 1, 8, and 9 months	Control diet	Inflammation	↓	Rivera-Huerta *et al.* 2017 [74]
		15.7% or 10% for 20 weeks	Control diet/cooked ham	Tumor number SCFAs *Desulfovibrio* *Bilophila*	↓ ↑ ↓ ↓	Fernández *et al.* 2019 [78]
	Mouse	7.5% for 4 weeks	Control diet cellulose	Luminal succinate Inflammation Tumor size Tumor number	↑ ↑ ↑ ↑	Tian *et al.* 2023 [92]
*Apc^Min/+^* mice (CRC)	Mouse	10% for 4 weeks	Control diet cellulose	Tumor size Tumor grade *E. coli pks+*	↑ ↑ ↑	Oliero *et al.* 2023 [105]
		10% for 14 weeks	HF diet	Tumor size	↑	Misikangas *et al.* 2008 [99]
		10% for 9, 12, or 15 weeks	HF diet	Tumor size Tumor number	↑ ↑	Pajari *et al.* 2003 [102]
*Apc^Min/+^* AOM (CRC)	Mouse	15% for 8 weeks	Inulin diet 5%	Tumor number α-diversity	↑ ↓	Moen *et al.* 2016 [103]
		5%, 10%, 15%, 20%	Control diet	Tumor number Tumor volume	↑ ↑	Yang *et al.* 2023 [104]
4-PAHs/DSS (CAC)	Mouse	10 g/L for 3 weeks	Water	Tumor number	No effect	Zaoui *et al.* 2024 [128]
Anastomotic leak	Mouse	10% for 2 weeks	Control diet cellulose	SCFAs Anastomotic healing	↑ ↑	Hajjar *et al.* 2021 [83]
Anastomotic leak (CRC)	Mouse	10% for 4 weeks	Control diet cellulose	SCFAs Anastomotic tumors Peritoneal tumors	↑ ↓ ↓	Hajjar *et al.* 2024 [86]

↑ increase; ↓ decrease.

ACF = aberrant crypt foci, AOM = azoxymethane, CAC = colitis-associated CRC, CRC = colorectal cancer, DMH = 1,2-dimethylhydrazine, HF = high-fat, HP = high-protein, IL = interleukin, LP = low-protein, SCFAs = short-chain fatty acids, 4-PAHs = 4-polycyclic aromatic hydrocarbons.

**Table 2.** Summary of clinical trials on inulin supplementation

Disease	Treatment	*N*	Amount and duration	Control group	Outcomes	Effect	Reference
CRC	Colectomy	20	6 g twice a day for 6 months	Maltodextrin powder	ACF number Proliferation Apoptosis	No effect No effect No effect	Limburg *et al.* 2011 [125]
CRC	Colectomy	19	(Synbiotic) 10 g/day for 12 weeks	Maltodextrin	Immune parameters	No significant effect	Roller *et al.* 2007 [127]
Polyps	Polypectomy	19	(Synbiotic) 10 g/day for 12 weeks	Maltodextrin	Immune parameters	No significant effect	
CRC	Colectomy	19	(Synbiotic) 12 g/day for 12 weeks	Placebo	*Bifidobacterium*	↑	Rafter *et al.* 2007 [129]
					*Lactobacillus* CRC markers Necrosis Genotoxicity	No effect No effect No effect No effect	
Polyps	Polypectomy	19	(Synbiotic) 12 g/day for 12 weeks	Placebo	*Lactobacillus* *Bifidobacterium* *C. perfringens* Genotoxicity Necrosis	↑ ↑ ↓ ↓ ↓	
FAP	J-pouch	9	(Synbiotic) 12 g/day for 4 weeks	Sulindac and Sulindac–inulin combination	Proliferation GST Cytotoxicity SCFAs Fecal pH	No effect No effect No effect No effect No effect	Friederich *et al.* 2011 [126]
FAP & UC	J-pouch	20	24 g/day for 3 weeks	Crossover study, placebo	*B. fragilis* Fecal pH SBAs Butyrate PDAI	↓ ↓ ↓ ↑ ↓	Welters *et al.* 2002 [89]
Gynecological cancer	Postoperative radiotherapy	20	(Mix inulin and FOS) 6 g twice daily for 3 weeks	Maltodextrin	Stool consistency	↑	Garcia-Peris *et al.* 2016 [133]
					Global health Symptoms	No effect No effect	
Breast cancer	Neoadjuvant chemotherapy	19	15 g/day for 21 days	Maltodextrin	Blood pressure Heart rate	↓ ↓	Becerril-Alarcón *et al.* 2019 [134]
					BMI	↑	
					BFP	No effect	
CRC	Surgery resection	85	6 g twice daily for 6 months	Placebo	ACF number Proliferation Apoptosis	Ongoing	NCT00335504
Myeloma	HSCT	100	(Mix inulin and FOS) 5 g/day for 28 days	Placebo	BMI Gut symptoms Infection	Ongoing	NCT05460013
Febrile neutropenia	Chemotherapy	120	12 g/day for 3 months	Placebo	Body temperature Neutrophil	Ongoing	NCT02544685

↑ increase; ↓ decrease.

*N* = number of participants, ACF = aberrant crypt foci, BFP = body fat percentage, BMI = body mass index, CRC = colorectal cancer, FAP = familial adenomatous polyposis, FOS = fructooligosaccharides, GST = glutathione S-transferase, HSCT = hematopoietic stem cell transplantation, PDAI = pouchitis disease activity index, SBAs = secondary bile acids, SCFAs = short-chain fatty acids, UC = ulcerative colitis.

## The good …

The primary prebiotic function of inulin is linked to stimulating the growth of beneficial bacteria, mostly strains from *Bifidobacterium* and *Lactobacillus* genera, and reducing the expansion of harmful bacteria [38, 39]. The composition of the gut microbiota has the potential to impact CRC carcinogenesis, notably through the production of pro- or anti-tumorigenic metabolites [40]. The bacterial enzymes β-glucuronidase, β-glucosidase, azoreductase, and nitroreductase, highly expressed in many *E. coli* and *Clostridium* species, convert pre-carcinogenic molecules into carcinogens and are therefore implicated in CRC development [41, 42]. *Bifidobacterium* and *Lactobacillus* species have been shown to suppress the activity of these pro-carcinogenic enzymes [43–45], which is consistent with the decrease in β-glucuronidase and β-glucosidase activity observed with inulin supplementation *in vivo* [46].

Bacterial fermentation of inulin in the colon produces beneficial metabolites such as the SCFAs acetate, propionate, and butyrate [47]. Inulin supplementation specifically was shown to promote the growth of *Clostridium* cluster XIVa butyrate producers [48]. Overall, butyrate has been linked most consistently with gut health, as it can limit the expansion of potentially pathogenic bacteria, decrease inflammation, regulate gene expression, increase the gut barrier function, and improve reabsorption by lowering colonic pH.

For example, butyrate activation of the peroxisome proliferator-activated receptor-γ (PPAR-γ) signaling pathway in colon epithelial cells induces metabolic β-oxidation, resulting in an increasingly anaerobic environment that limits the growth of facultative aerobes such as *E. coli* and *Salmonella enterica* [49]. PPAR-γ activation has long been associated with the inhibition of pro-inflammatory cytokines and patients with PPAR-γ-positive colorectal tumors had significantly lower overall and CRC-specific mortality [50, 51]. Butyrate may also protect against cancer by regulating the expression and activity of numerous proteins involved in cancer initiation and cancer progression [52, 53]. Notably, butyrate is a potent inhibitor of histone deacetylases [54], which are shown to be pro-proliferative in CRC due to their transcriptional repression of cell cycle inhibitors [55]. In addition, butyrate improves the gut barrier function by increasing the expression of tight junction proteins [56], the release of antimicrobial peptides [57], and the thickness of the mucus layer [58]. Finally, SCFAs improve gut health by lowering the colon pH, which increases the absorption of minerals such as iron [59] and the reabsorption of CRC-promoting bile acids [60].

Several studies using animal models report potential beneficial effects of inulin supplementation when given in doses ranging from 20 to 157 g/kg chow (Table 1). Most of these studies use a carcinogenic compound to initiate the tumor event, such as azoxymethane (AOM) or 1,2-dimethylhydrazine (DMH), and report a decrease in the number of ACF [61–74] upon inulin supplementation. One study proposes that the lower ACF counts observed in inulin-treated rats in a DMH model of early colon carcinogenesis are due to a decrease in tumorigenic secondary bile acids [46]. As inulin fermentation and SCFA production increase, the pH of the colon decreases and the bacterial enzyme 7-α-dehydroxylase, responsible for the final step in secondary bile acid synthesis, is inhibited [46]. However, it must be noted that, while ACF are commonly considered as precursors for colon cancer, they do not always develop into tumors [75], rendering studies based solely on these parameters somewhat inconclusive. Some studies also report a decrease in DNA damage [70] and/or in the number of tumors [76–79] (Table 1). In addition, a study focusing on the cancer-preventive effects of inulin during early carcinogenic events reported an increase in apoptotic cells [80], which is associated with reduced tumorigenesis, as a balance is maintained between newly formed cells and surviving cells. This is in agreement with findings that the low-fiber Western diet is associated with a decrease in apoptotic cells accompanied by an increase in carcinogenesis [81]. Inulin was also shown to have anti-metastatic effects in a mouse orthotopic CT-26 implantation model of CRC progression, with lower tumor count and decreased tumor weight in the liver in comparison with cellulose or a mixed-fiber diet [82]. Among these groups, inulin had the strongest correlation between changes in gut microbiota composition, SCFA levels, markers of epithelial to mesenchymal transition, and metastasis parameters, indicating that the role of inulin in preventing CRC metastasis to the liver may be microbiota-mediated [82].

Besides its direct effect on tumor initiation and progression, preoperative inulin supplementation has been proposed as a way to improve healing following CRC surgery due to its inhibition of collagen degradation and promotion of colonocyte proliferation and colonic re-epithelialization following tumor resection [83]. In a murine model of colonic anastomosis, a 2-week treatment with inulin before surgery stimulated colonic repair and prevented anastomotic leaks [83]. The importance of preventing anastomotic leaks and reinforcing gut barrier integrity and recovery after surgery is demonstrated by the strong association between the gut microbiota, anastomotic leak, and clinical and oncological outcomes [84]. Given its ability to reinforce the gut barrier, inulin supplementation may decrease the dissemination of cancer cells and the potential for CRC metastasis in cases of poor postoperative healing [85, 86]. In fact, mice that received inulin followed by colonic surgery and a direct inoculation of CT-26 CRC cells by enema had smaller tumors at the site of intestinal resection as well as a decreased peritoneal tumor count and lower tumor index [86].

In humans, short-term inulin supplementation has also been tested as a strategy to enhance healing after intestinal resection and ileo-anal anastomosis or J-pouch—a common surgical procedure for ulcerative colitis and FAP patients [87, 88]. In patients with ileal pouch inflammation, a placebo-controlled trial studied the effect of 24 g of inulin daily and showed increased concentrations of butyrate, diminished concentrations of bile acids in feces, and decreased abundance of *B. fragilis*, which may be of relevance to patients colonized with enterotoxigenic *B. fragilis* [89]. Clinically, inulin supplementation led to lower levels of mucosal inflammation both endoscopically and histologically [89]. Such studies have paved the way for targeted treatments before surgery to enhance wound healing, which is central to preventing cancer recurrence, improving survival, and maintaining quality of life [90].

To summarize, *in vitro*, preclinical, and human studies have shown that inulin has beneficial effects, including reduced ACF count, decreased inflammation, and protection against DNA damage, that are associated with a lower risk of colorectal cancer. These beneficial outcomes are largely mediated by the effects of inulin on the gut microbiota composition and production of butyrate.

## The bad …

Despite the promising results shown with inulin supplementation and a general consensus surrounding the benefits of inulin for gut health, more recent studies in mice show that inulin may produce harmful effects. These include an increased production of tumorigenic microbial metabolites, including secondary bile acids [91] and succinate—a key intermediate metabolite in SCFA production [92]. Succinate has been shown to promote tumor progression and metastasis through the activation of tumor-associated macrophages and the inhibition of CD8^+^ T-lymphocyte infiltration into the tumor microenvironment [93, 94].

Furthermore, both primary bile acids secreted by the liver and secondary bile acids resulting from the transformation of primary bile acids by gut bacteria have been linked to CRC [95]. Notably, bile acids are capable of damaging the colonic epithelium and increasing the production of reactive oxygen species that alter DNA and induce apoptosis resistance [96].

While inulin has been shown to exacerbate colitis [97, 98] and colitis-associated CRC [92], it has also been linked to non-colitis-associated CRC tumorigenesis [99, 100]. At the preclinical stage, inulin has been shown to promote the growth of genotoxin-producing bacteria, induce microbial-mediated immunosuppression, and drive the progression of colonic adenomas towards malignancy [99, 100]. Studies in the *Apc^Min/+^* mice model harboring a non-sense mutation in the *Apc* gene analogous to that in FAP patients [101] found that inulin supplementation stimulates tumor cell growth. Two studies carried out by Misikangas *et al.* [99] and Pajari *et al.* [102] showed an increase in tumor burden when *Apc^Min/+^* mice were given long-term inulin supplementation. Another study using *Apc^Min/+^* mice and injection with AOM, a mutagenic compound, compared three different diets containing inulin, cellulose, or brewers spent grain (BSG) [103]. The three diets resulted in an increase in tumor burden in a dose-dependent manner following an 8-week intervention, suggesting that the beneficial impacts of inulin are mitigated at higher doses. Similar data also showed that inulin promoted tumorigenesis in *Apc^Min/+^* mice in a dose-dependent fashion [104]. More recently, we showed that a 4-week inulin intervention in *Apc^Min/+^* mice colonized with the *pks+ E. coli* NC101 murine strain resulted in an expansion of *E. coli* NC101 and enhanced tumorigenicity [105]. *Escherichia coli* isolates from patient colonic tumors were also shown to have higher *pks* island expression levels [106]. Crucially, the *pks* island and encoded colibactin synthesizing enzymes have been linked with a unique mutational profile in CRC, indicating an active role in CRC mutagenesis [107].

A study in mice revealed that inulin exacerbates acute colitis [108]. An increase in butyrate-producing bacteria and cecal butyrate levels, potentially due to impaired butyrate oxidation [109], was also reported [108]. Interestingly, while low concentrations of butyrate (0.1 mM) stimulated colonic epithelial stem/progenitor cell proliferation *in vitro*, concentrations of >1 mM suppressed proliferation [110]. Even as normal butyrate concentrations in the colon can exceed 70 mM [111], an intact mucus layer prohibits direct contact with the colonic epithelium. This is not true in the case of colitis, in which a weakened mucus barrier [112] may expose the mucosal epithelium to excess butyrate.

Recent studies have highlighted that the effects of diet and prebiotics vary significantly according to the gut microbiota composition [113–115]. When bacteria capable of fermenting inulin are absent, inulin remains unfermented in the gut and can trigger inflammation. Unfermented β-fructans (fructooligosaccharides and inulin) exacerbated symptoms and even increased inflammation in a subset of patients with active ulcerative colitis [116]. Inulin was also shown to bind to immune cells, notably monocytes and macrophages, via the toll-like receptor (TLR)-2 [116] and TLR-4 [117]. TLRs play crucial roles in the innate immune system by recognizing pathogen-associated molecular patterns derived from various microbes such as lipopolysaccharide (LPS). For instance, TLR4 is involved in pro-inflammatory signaling and is normally stimulated by the LPS of gram-negative bacteria [118]. In addition, inulin has been found to stimulate type 2 inflammation, linked to atopic dermatitis (eczema), allergic rhinosinusitis, and some types of asthma through a shift in microbial composition and an increased release of bile acids [98]. Type 2 inflammation plays a major role in intestinal homeostasis, as well as in deleterious chronic inflammatory conditions, such as IBD [119].

In summary, studies in preclinical animal models indicate that inulin supplementation aggravates colitis and promotes the development of sporadic CRC. Meanwhile, in humans, inulin appears to aggravate colitis in a subset of IBD patients, but its impact on non-colitis-associated CRC remains to be explored. A recent case study highlights long-term daily inulin consumption as a potential contributing factor in a patient’s metastatic CRC [120]. However, while thought-provoking, this evidence remains anecdotal and the patient’s diagnosis cannot be definitively linked to their inulin supplementation. Further placebo-controlled, long-term clinical studies or retrospective clinical analyses examining the effects of inulin supplementation on sporadic CRC development are needed to clarify in which cases long-term inulin supplementation may help to promote or prevent CRC development.

## … and the unhelpful

In most clinical studies, inulin supplementation (ranging from 10 to 15 g/day during 1–6 months) failed to convincingly demonstrate a preventive effect on CRC development (Table 2). In a prospective cohort study of 53,700 participants, no association was found between prebiotic intake and prevention of CRC risk [121]. Furthermore, in two systematic reviews and in a multicenter longitudinal study, inulin was ineffective in preventing colorectal neoplasia [122–124]. A study by Limburg *et al*. [125] did not show any beneficial effects of 6 g of inulin twice per day for 6 months on rectal ACF. However, the study had limited power due to a small sample size. In a study by Friederich *et al*. [126], 12 g of inulin per day combined with probiotic supplementation for 4 weeks did not significantly lower the risk of adenoma development in the J-pouch of patients with FAP. Finally, a study by Roller *et al.* [127] in patients with CRC or polyps showed that a 12-week regimen of 10 g of daily inulin in combination with probiotics only had minor effects on selected immune parameters.

Even if inulin supplementation modulates the gut microbiota and increases concentrations of SCFAs, these changes may be insufficient to provide a better disease outcome. In mice, inulin supplementation failed to reverse the effects of a potentially carcinogenic compound, namely polycyclic aromatic hydrocarbon (4-PAH), produced by the high-heat cooking of meat [128]. While 4-PAHs were shown to promote tumorigenesis in the context of dextran sodium sulfate (DSS)-induced intestinal inflammation, the presence of inulin did not have any effect on tumor count reduction or histological parameters [128].

In humans, a placebo-controlled clinical trial used a combination of oligofructose-enriched inulin and probiotics (*Bifidobacterium lactis* Bb12 and *Lactobacillus rhamnosus* GG) in CRC and polypectomized patients [129]. The combination induced a favorable change in the gut microbiota (increase in *Bifidobacterium* and *Lactobacillus* species and decrease in *Clostridium perfringens)* without affecting the patients’ overall CRC parameters. Only patients with polypectomies exhibited reduced proliferation, lower fecal water toxicity, and improved inflammatory markers [129]. Furthermore, a recent systematic review of trials examining inulin supplementation in healthy, overweight, or IBD participants revealed a significant increase in the *Bifidobacterium* genus, which nevertheless failed to enhance SCFA levels [130]. In these studies, the absence of a beneficial effect from inulin supplementation may be explained by limited power, inadequate form of administration, or insufficient dose and/or duration. Nevertheless, due to the digestive discomfort experienced by some participants and the detrimental results of high-dose inulin *in vivo*, there is a limited margin for increasing inulin dose concentrations in order to achieve significant effects.

Overall, these studies reveal the need to better define relevant microbial and clinical markers to predict responses to inulin supplementation in regard to cancer initiation and progression.

## Perspectives

While studies show that long-term inulin supplementation may not be helpful, relatively short-term (<1 month) inulin supplementation in conjunction with targeted cancer treatments has yielded positive results in the enhancement of treatment response. A recent study advanced the idea of combining a prebiotic-based drug delivery system with the oral administration of chemotherapeutic agents for CRC at lower doses than would be required if administered systemically [131]. Given that inulin has previously been classified as an optimal drug carrier due to its flexible structure, stability, and biodegradability [132], an inulin-based delivery system would allow a drug to resist digestion and absorption in the small intestine prior to its targeted release in the colon. This type of approach using a xylan polysaccharide-based capsule successfully delivered oral capecitabine to CRC tumors, promoted microbiota-mediated antitumor responses, and increased the abundance of select probiotic bacteria [131].

In other cancers, short-term inulin supplementation during therapeutic treatments such as surgery, radiation therapy, and chemotherapy has been shown to yield positive results, enhance response to treatment, alleviate symptoms, shorten convalescence, and improve quality of life. For example, in the context of gynecological cancer, patients who received inulin and fructooligosaccharides before and during radiation therapy presented an improved stool consistency compared with those receiving placebo [133]. During breast cancer-related chemotherapy, 15 g of inulin for 21 days during treatments lowered systolic blood pressure and increased body mass index [134]. Furthermore, inulin has been employed as an adjuvant for influenza vaccination and allergy treatment to enhance the immune response [135–138], and recent research showed that inulin supplementation could improve cancer responses to immunotherapy treatments [138]. The success of inulin supplementation in conjunction with treatments for various types of cancers highlights how inulin may be used in the context of CRC.

Overall, short-term inulin supplementation presents interesting opportunities in various aspects of CRC treatment, as it may improve surgical healing, enhance radiation therapy tolerance, and improve immune responses to both systemic and targeted chemotherapy treatments, all of which necessitate further exploration in clinical trials.

## Conclusions

To summarize, inulin supplementation has shown potential benefits in animal and human studies. Short-term inulin supplementation during cancer therapy can enhance responses to conventional therapy, relieve symptoms, shorten recovery, and improve surgical healing and quality of life. However, inulin can also drive intestinal inflammation, increase the proliferation of genotoxin-producing bacteria, and aggravate IBD and colitis-associated CRC. Both positive and negative effects of inulin are dependent on the initial gut microbiota composition and further studies are required to better identify patients who may benefit from or be harmed by inulin supplementation, based on whether they harbor specific microbial taxa capable of degrading inulin. Given the potential negative effects of high-dose inulin, a better mechanistic understanding of its role in regulating inflammation is needed, especially in patients with IBD and colitis-associated CRC. Finally, it is important to note that the negative effects of inulin have been observed in the context of highly purified supplements, the physiochemical properties of which may differ from inulin that is naturally present within the food matrix. Ultimately, while purified inulin supplementation may provide benefits as part of a treatment regimen, it is likely insufficient to completely overturn the harmful effects of a continued Western-style diet.

## Authors’ Contributions

M.O., A.A.A., C.M., and M.M.S. contributed to validation, and wrote and reviewed the manuscript. M.M.S. additionally contributed to supervision, resources, and funding acquisition of the study. All authors read and approved the final manuscript.
